# Effects of N-acetyl-L-cysteine on sperm quality during chilled storage of Maguan hornless goat semen

**DOI:** 10.1371/journal.pone.0347756

**Published:** 2026-04-21

**Authors:** Haoran Xu, Maosheng Cao, Qingwei Wang, Yonghong Ju, Xiaodong Wang, Qing Liu, Tingji Zeng, Xiang Chen

**Affiliations:** 1 Key Laboratory of Animal Genetics, Breeding and Reproduction in the Plateau Mountainous Region, Ministry of Education, Guizhou University, Guiyang, China; 2 Key Laboratory of Animal Genetics, Breeding and Reproduction of Guizhou Province, Guiyang, China; 3 College of Animal Science, Guizhou University, Guiyang, China; Zagazig University, EGYPT

## Abstract

The Maguan hornless goat is a valuable indigenous goat breed in Yunnan Province, China. As a rare and endangered genetic resource, its population protection and breeding are challenged by numerous challenges, particularly during artificial insemination procedures. During chilled storage, sperm generate excessive levels of reactive oxygen species (ROS), which disrupt the integrity of the sperm plasma membrane, damage the celluar structures, and lead to a decline in semen quality. This study investigated the effects of different concentrations (0, 5, 7, 9 mM) of N-acetylcysteine (NAC) on the chilled storage of semen collected from Maguan hornless goats. Semen was collected from four healthy rams with normal reproductive performance. The different concentrations of NAC were added to the semen diluent, and the samples were diluted 10-fold prior to storage at 4 °C for up to 72 h. After defining storage intervals, sperm kinetics, antioxidant gene transcription, and oxidative stress–related enzymatic activity were analyzed. Results showed that sperm samples extended in diluent supplemented with 7 mM NAC and subjected to 72 h of chilled storage at 4 ℃ exhibited improved antioxidant levels. This approach also reduced sperm apoptosis, enhanced membrane integrity and mitochondrial membrane potential, and suppressed the expression of pro-apoptotic genes (*BAX, Caspase 3*) while upregulating that of antioxidant genes (*GPX4, GPX1*). The results suggest NAC supports semen chilled storage in endangered species by enhancing antioxidant capacity and inhibiting cell death-related pathways. This study provides insights into the application of reproductive biotechnology in small ruminants.

## Introduction

The Maguan hornless goat is an endangered indigenous breed native to Maguan County in Yunnan Province, China, characterized by early sexual maturity, high prolificacy, and strong tolerance to environmental stress. However, due to its limited population size and scattered distribution, it faces serious risks of genetic drift and breeding decline. Therefore, efficient reproductive biotechnology approaches are required to enhance population expansion and improve the utilization efficiency of elite sires. The quality of sperm preservation is an important factor affecting reproductive efficiency, as it influences the success of artificial insemination and the effectiveness of genetic resource conservation. Semen preservation can generally be categorized into room-temperature liquid storage, chilled storage, and cryopreservation (freezing) [1]. Chilled sperm storage is an important component of modern animal production systems [2]. It transforms breeding from a traditional technique constrained by time and geography into a scientific approach that can be precisely designed, efficiently disseminated, and offers with long-term protection [3]. Goat semen can be preserved for up to three days using chilled storage. During chilled storage, the metabolic rate and oxygen consumption of sperm cells are significantly reduced [4], thereby maintaining fertilization capacity to a certain extent. However, during chilled storage, semen is subjected to low-temperature stress, leading to excessive production of reactive oxygen species (ROS), which ultimately compromises sperm motility and overall semen quality.

N-acetylcysteine (NAC) is a small molecule antioxidant naturally occurring as a crystalline compound [5]. It is a derivative of L-cysteine and a precursor of reduced glutathione (GSH) [6], with antioxidant and free radical scavenging effects. The use of NAC in animal reproduction has attracted increasing scientific interest [7], with previous studies demonstrating the protective effects of adding antioxidants to semen diluent to alleviate oxidative stress during cryopreservation. Supplementation with NAC attenuates oxidative stress, improves glutathione-related redox imbalance, and alleviates oxidative stress-associated cellular damage [8]. Semen exposure to H_2_O_2_ can significantly reduce sperm motility, increase the incidence of abnormal morphology, and damage plasma membrane integrity; whereas NAC treatment can prevent sperm deterioration, offset oxidative damage, and improve sperm motility, morphology, and chromatin and membrane integrity [9]. Adding NAC to chicken semen has been shown to improve sperm motility, the percentage of sperm exhibiting progressive and rapid motility, and increase average path (VAP), straight-line velocity (VSL), and curvilinear velocity (VCL) [10]. Moreover, chronic exposure of mice to lead has been reported to reduced sperm motility, viability, and motor performance, and increased expression of apoptotic genes, *BAX* and *Caspase 3*. NAC supplementation improved semen quality, enhanced antioxidant balance, and reduced apoptosis [11]. Despite numerous reports on the antioxidant effects of NAC on sperm preservation, the underlying mechanisms have not been fully elucidated.

Although several studies have investigated the antioxidant effects of N-acetylcysteine (NAC) in reproductive systems and semen preservation in small ruminants, information regarding its application in goat semen diluents during chilled storage remains limited. Furthermore, the molecular mechanisms underlying NAC-mediated protection, particularly its role in regulating antioxidant and apoptosis-related pathways during chilled storage, remain insufficiently understood. Therefore, the present study aimed to address these gaps by systematically evaluating the effects of NAC supplementation on sperm quality and associated molecular responses in Maguan hornless goat semen during chilled storage. The findings of this study may provide valuable insights for optimizing semen preservation strategies in goats and may support future applications in artificial insemination programs [12].

## Materials and methods

### Experimental animals

Four healthy Maguan hornless goat rams aged 2–3 years were used in this experiment. The animals were obtained from the Teaching and Experimental Base of Guizhou University. They were housed in 2 separate pens with *ad libitum* access to food and water. The experimental protocol was approved by the Animal Ethics Review Committee of Guizhou University (Approval No. EAE-GZU-2025-E080).

### Semen collection

Semen was collected from four rams using an artificial vagina. A total of 10 ejaculates were obtained throughout the experimental period [13]. Immediately after collection, semen samples were evaluated for color, volume, and viscosity, and sperm motility was assessed microscopically. Only ejaculates with sperm motility exceeding 70% were included in the study [14]. After the initial quality assessment, qualified semen samples were pooled by mixing equal volumes to minimize individual variation and then used for the storage experiments and parameter measurements.

### Semen processing

The commercial diluent, designated as diluent I without antioxidants, was purchased from Beijing Tianyuan Aorui Biotechnology Co., Ltd. (Beijing, China). NAC was added to the diluent I in the experimental groups at concentrations of 5, 7, and 9 mM, while no NAC was added in the semen diluent in the control group. The samples were stored at 4 °C for 72 h and gently mixed every 12 h to minimize sperm sedimentation without introducing mechanical stress. Sperm motility was assessed at 24-h intervals, and sperm antioxidant enzyme activity and plasma membrane integrity were evaluated after 72 h of storage.

### Semen quality testing

Semen quality was evaluated at 24-h intervals over three consecutive days. Before evaluation, the slide and microscope stage were preheated to 37 °C. A 5 μL aliquot of semen was placed on the slide and covered with a coverslip to avoid bubble formation. Each sample was incubated for 2 min at 37 °C [15]. Sperm motility parameters were assessed using a computer-assisted sperm analysis system (CASA; SJ-TMDI210, Nanning Songjing Tianlun Biotechnology Co., Ltd., Nanning, China) at 10 × magnification. The system was configured with an acquisition frame rate of 60 fps. For each sample, a minimum of six randomly selected microscopic fields or 200 motile spermatozoa were analyzed to ensure statistical robustness. The CASA settings included a cell size range of 2–30 μm, a calibration scale of 192 pixels/100 μm, and a motility threshold of 20 μm/s. The recorded parameters included sperm viability, total motility, average path velocity (VAP), straight-line velocity (VSL), curvilinear velocity (VCL), wobble (WOB), linearity (LIN), and straightness (STR). At least three biological replicates were analyzed per experimental group.

### Detection of sperm antioxidant capacity

Sperm total antioxidant capacity (A015-1) [16], trace reduced glutathione (A006-2–1), ROS (CA1410), superoxide dismutase (SOD; A001-3), GSH-Px (A005-1), CAT (A007-1–1), malondialdehyde (MDA; BC0025) and other reagents were purchased from Nanjing Jiancheng Bioengineering Research Institute (Nanjing, China) and Beijing Solaibao Technology Co., Ltd. (Beijing, China).

### Sperm cell structure detection

#### Detection of sperm ROS, cell energy level, and mitochondrial membrane potential.

Intracellular ROS were quantified using a DCFH-DA probe (1–10 μmol/L, serum-free). [17]. Following sample preparation, sperm cells were incubated with the probe solution (≥1 mL per well) at 37°C for 15−60 min and subsequently washed three times to remove excess extracellular probe. Sperm ATP content was measured using an enhanced ATP detection kit (Beyotime Biotechnology, Shanghai, China; S0027). Mitochondrial membrane potential was evaluated using the JC-1 reagent (Solaibao Technology, Beijing, China; M8650). Semen (30 μL) was diluted in buffer (0.5 mL), combined with equal volumes of JC-1 solution, and incubated (37 °C, 20 min) [18]. After centrifugation (600 × *g*, 5 min, 4 °C) and dual buffer washes, pellets were resuspended (0.5 mL) for fluorometric quantification via a microplate reader [19].

#### Sperm plasma membrane and acrosome integrity assessment.

Membrane integrity was assessed by a hypotonic swelling assay using a commercial kit (Beijing Solaibao Technology Co., Ltd., Cat. No. G2580). Pre-warmed hypotonic solution (1 mL, 37 °C, 5 min) was combined with semen samples (0.1 mL) and maintained at 37 °C for 45 min under gentle agitation. Subsequently, the proportion of tail-coiled spermatozoa was quantified via microscopic examination. Acrosome status was evaluated using fluorescein-conjugated peanut agglutinin (PNA-FITC) staining following the manufacturer#39;s instructions (Genmed, Plymouth, MN, USA) [20].

#### Detection of sperm apoptosis.

Apoptotic spermatozoa were quantified using Annexin V-FITC/PI dual-staining (Shanghai Beyotime Biotechnology, Shanghai, China. C1062L). Semen samples were centrifuged (1000 × g, 5 min), washed with PBS, centrifuged again, and resuspended in binding buffer (195 μL). Sequential staining with Annexin V-FITC (5 μL) and PI (10 μL) was followed by incubation in the dark at 20–25 °C for 10–20 min. After incubation, samples were centrifuged, resuspended (100 μL), and 20 μL aliquots were analyzed using fluorescence microscopy (Nikon Eclipse Ti-U). Green fluorescence (FITC channel: λex = 488 nm, λem = 525 nm) indicated Annexin V-positive cells, while red fluorescence (PI channel: λex = 535 nm, λem = 615 nm) indicated membrane-compromised cells. A minimum of 200 spermatozoa from 3–5 randomly selected fields were analyzed per sample. Apoptotic cells were categorized into early apoptotic (Annexin V-FITC ⁺ /PI⁻) or late apoptotic/necrotic (Annexin V-FITC ⁺ /PI⁺), with rates computed as percentages of total counted cells.

### RNA extraction and quantitative real-time polymerase chain reaction (qRT-PCR)

Total RNA was extracted using an RNA isolation kit (Sigma-Aldrich, UK) and reverse transcribed using PrimeScript RT reagent with gDNA Eraser (Takara Bio, Otsu, Japan) according to the manufacturer#39;s instructions [21]. qRT-PCR reactions were prepared in a master mix containing SYBR Green Master Mix (10 μL, Takara Bio, Japan), forward and reverse primers (0.5 μL each), template cDNA (5 ng per reaction), and nuclease-free water to a final volume of 20 μL. The total volume of the master mix was 20 μL. Thermal cycling parameters included initial denaturation (95°C, 2 min), followed by 40 amplification cycles of denaturation (95°C, 15 s), and combined annealing/extension (60°C, 30 s). Gene expression levels were normalized to GAPDH as the endogenous control and calculated using the 2^−ΔΔCt^ comparative method. Primers were synthesized by Shanghai Shenggong Biological Engineering Co. Ltd. (Shanghai, China). See Table 1 for details of primer sequences.

**Table 1:** Primer sequence information.

Gene	Primer information (5’ → 3’)	Product length (bp)	Annealing temperature (° C)
*BAX*	F: GGGTAGGGAGGAGTGGTCTT	175	60
	R: TTAACTCACCGCTGACCCAC		
*GPX1*	F: CAGGAAAATGCCAAGAACGAG	82	60
	R: GCATGAAATTGGGCTCGAAC		
*GPX4*	F: TGAGGCAAGACTGACGTAAAC	135	60
	R: TTTGATCTCCGCATTACTCCC		
*Caspase 3*	F: AGACCATAGCAAAAGGAGCAG	169	60
	R: TGGGTTTTCCAGTCAGACTTC		
*GAPDH*	F: TGGAGAAACCTGCCAAGTATG	127	60
	R: TGAGTGTCGCTGTTGAAGTC		

### Statistical analysis

Statistical evaluation was performed using SPSS version 27.0 (IBM Corp., Armonk, NY, USA). Each experiment included a minimum of three independent biological replicates, and the data were expressed as the mean ± standard deviation (SD). Prior to analysis, the normality of the data distribution was assessed using the Shapiro–Wilk test, and homogeneity of variances was evaluated using Levene’s test. Differences among multiple treatment groups were analyzed using one-way analysis of variance (ANOVA).When a significant effect was detected, Duncan’s multiple range test was applied for post-hoc comparisons. Statistical significance was set at *P < 0.05,** *P* < 0.01, and ***P < 0.001.

## Results

### Effect of different concentrations of NAC on sperm motility after 24 h of chilled storage

**Fig 1:**
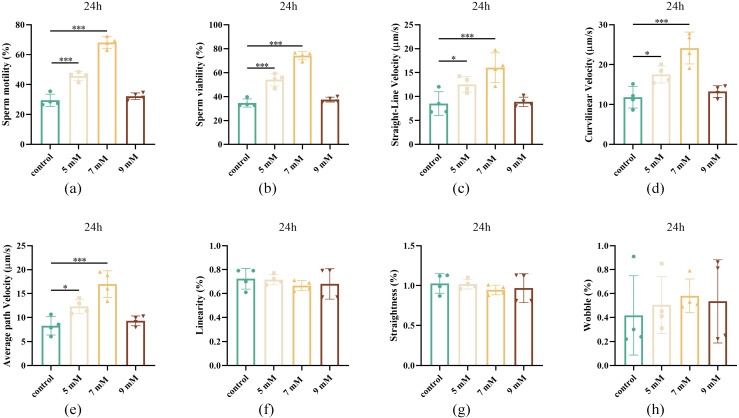
Effect of different concentrations of NAC on semen quality after 24 h of chilled storage (a) Sperm motility of the control, 5, 7, and 9 mM NAC groups after storage at 4℃ for 24 h. (b) Sperm viability of the control, 5, 7, and 9 mM NAC groups after storage at 4℃ for 24 h. (c) Straight-line velocity of the control, 5, 7, and 9 mM NAC groups after storage at 4℃ for 24 h. (d) Curvilinear velocity of the control, 5, 7, and 9 mM NAC groups after storage at 4℃ for 24 h. (e) Average path velocity of the control, 5, 7, and 9 mM NAC groups after storage at 4℃ for 24 h. (f) Linearity of the control, 5, 7, and 9 mM NAC groups after storage at 4℃ for 24 h. (g) Straightness of the control, 5, 7, and 9 mM NAC groups after storage at 4℃ for 24 h. (h) Wobble of the control, 5, 7, and 9 mM NAC groups after storage at 4℃ for 24 h.

To investigate the effects of different concentrations of NAC on sperm quality after 24 h of chilled storage, a series of concentration groups were evaluated. As shown in Fig 1, after 24 h, the 7 mM NAC group showed a significantly improved sperm viability and motility, average path velocity (VAP), straight-line velocity (VSL), curvilinear velocity (VCL) compared with the control group (*P* ＜ 0.001, Fig 1a-e). However, the linearity (LIN) as well as straightness (STR) and wobble (WOB), did not differ significantly (*P* > 0.05, Fig 1f-h). Therefore, after 24 h of chilled storage, NAC significantly improved the overall sperm motility performance.

### Effect of different NAC concentrations on sperm motility after 48 h of chilled storage

**Fig 2:**
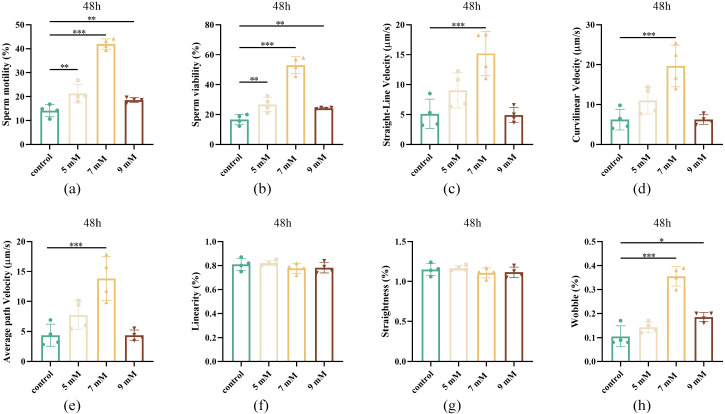
Effect of different concentrations of NAC on semen quality after 48 h of chilled storage (a) Sperm motility of the control, 5, 7, and 9 mM NAC groups after storage at 4℃ for 48 h. (b) Sperm viability of the control, 5, 7, and 9 mM NAC groups after storage at 4℃ for 48 h. (c) Straight-line velocity of the control, 5, 7, and 9 mM NAC groups after storage at 4℃ for 48 h. (d) Curvilinear velocity of the control, 5, 7, and 9 mM NAC groups after storage at 4℃ for 48 h. (e) Average path velocity of the control, 5, 7, and 9 mM NAC groups after storage at 4℃ for 48 h. (f) Linearity of the control, 5, 7, and 9 mM NAC groups after storage at 4℃ for 48 h. (g) Straightness of the control, 5, 7, and 9 mM NAC groups after storage at 4℃ for 48 h. (h) Wobble of the control, 5, 7, and 9 mM NAC groups after storage at 4℃ for 48 h.

To evaluate the prolonged effects of different NAC concentrations after 48 h of chilled storage, a series of concentration groups were assessed. Following 48 h of chilled storage, the 7 mM NAC group exhibited a significantly higher sperm viability, sperm motility, straight-line velocity (VSL), curvilinear velocity (VCL), average path velocity (VAP), and wobble (WOB) compared to the control group (*P* < 0.001, Fig 2a-e and 2h).

### Effect of different concentrations of NAC on sperm motility after 72 h of chilled storage

**Fig 3:**
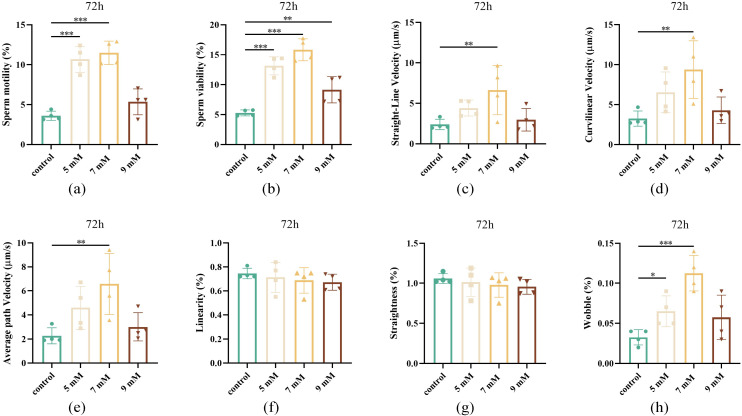
Effect of different concentrations of NAC on semen quality after 72 h of chilled storage (a) Sperm motility of the control, 5, 7, and 9 mM NAC groups after storage at 4 ℃ for 72 h. (b) Sperm viability of the control, 5, 7, and 9 mM NAC groups after storage at 4 ℃ for 72 h. (c) Straight-line velocity of the control, 5, 7, and 9 mM NAC groups after storage at 4 ℃ for 72 h. (d) Curvilinear velocity of the control, 5, 7, and 9 mM NAC groups after storage at 4 ℃ for 72 h. (e) Average path velocity of the control, 5, 7, and 9 mM NAC groups after storage at 4 ℃ for 72 h. (f) Linearity of the control, 5, 7, and 9 mM NAC groups after storage at 4 ℃ for 72 h. (g) Straightness of the control, 5, 7, and 9 mM NAC groups after storage at 4 ℃ for 72 h. (h) Wobble of the control, 5, 7, and 9 mM NAC groups after storage at 4 ℃ for 72 h.

The effects of different NAC concentrations on sperm quality parameters following 72 h of chilled storage were examined. As shown in Fig 3, the 7 mM NAC group exhibited superior sperm motility and sperm viability compared to the control group (*P* < 0.001, Fig 3a and 3b). Additionally, sperm at this concentration exhibited significantly elevated straight-line velocity (VSL), curvilinear velocity (VCL), average path velocity (VAP) (*P* < 0.01, Fig 3c-e), along with enhanced linearity (LIN) (*P* < 0.001, Fig 3f). The 5 mM NAC treatment also improved the wobble (WOB) relative to the control group (*P* < 0.05, Fig 3h). In summary, diluent supplementation with NAC markedly improved sperm motility across all treatment groups, with the 7 mM concentration yielding optimal results.

### Effect of different NAC concentrations on the antioxidant level of sperm after chilled storage for 72 h

**Fig 4:**
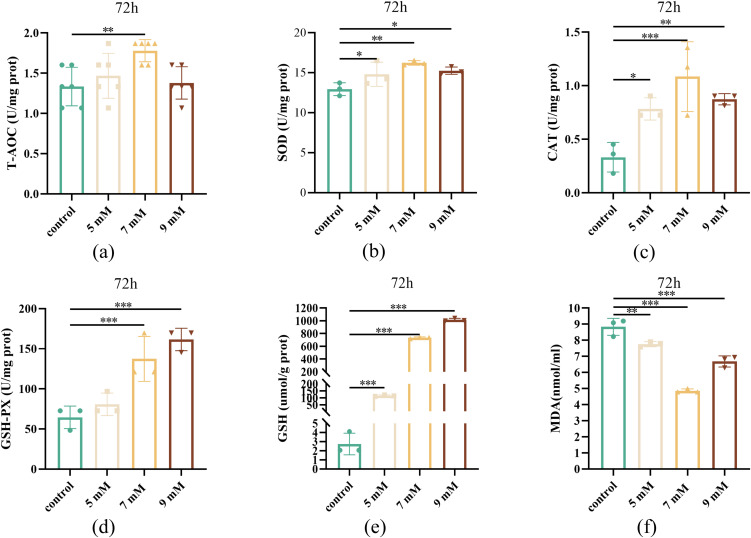
Effect of different concentrations of NAC on antioxidant enzyme activity of sperm after 72 h of chilled storage (a) Total antioxidant capacity of sperm in the control, 5, 7, and 9 mM NAC groups after storage for 72 h at 4 ℃. (b) SOD activity of sperm in the control, 5, 7, and 9 mM NAC groups after storage for 72 h at 4 ℃. (c) CAT activity of sperm in the control, 5, 7, and 9 mM NAC groups after storage for 72 h at 4 ℃. (d) GSH-Px activity of sperm in the control, 5, 7, and 9 mM NAC groups after storage at 72 h at 4 ℃. (e) GSH content of sperm in the control, 5, 7, and 9 mM NAC groups after storage at 72 h at 4 ℃. (f) MDA content of sperm in the control, 5, 7, and 9 mM NAC groups after storage for 72 h at 4 ℃.

During practical production, sperm chilled storage usually takes 48–72 h [22]. Therefore, the subsequent experiment in this study mainly focused on the effect of NAC on Maguan hornless goat semen quality after chilled storage for 72 h. As shown in Fig 4, the total antioxidant capacity in the 7 mM NAC group was the highest, which was also significantly higher than that in the control group (Fig 4a). The SOD activity in the 5 and 9 mM NAC groups was significantly higher than that in the control group (*P* < 0.05, Fig 4b). Notably, the 7 mM NAC group exhibited an even greater increase in SOD activity compared with the control group (*P* < 0.01, Fig 4b). The CAT activity in the 7 mM treatment group was substantially elevated compared to the control group (*P* < 0.001, Fig 4c), and the GSH-Px activity in the 7 and 9 mM NAC groups was significantly higher than that in the control group (*P* < 0.001, Fig 4d). The GSH content in the 5, 7, and 9 mM NAC groups was significantly higher than that in the control group (*P* < 0.001, Fig 4e), while the MDA content in the 7 and 9 mM NAC groups was significantly reduced compared to the control group (*P* < 0.001, Fig 4f). In conclusion, NAC significantly enhances sperm antioxidant capacity and reduces oxidative stress damage during chilled storage.

### Effect of different NAC concentrations on sperm structure after 72 h of chilled storage

#### Effects of NAC on ROS, cell energy level, and mitochondrial membrane potential of sperm after chilled storage for 72 h.

**Fig 5:**
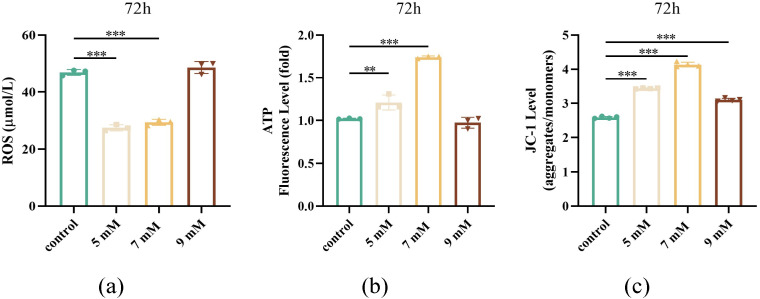
Effects of different concentrations of NAC on sperm reactive oxygen species, cell energy level, and mitochondrial membrane potential (a) ROS levels in the control, 5, 7, and 9 mM NAC groups after storage for 72 h at 4 ℃. (b) The levels of ATP in the control, 5, 7, and 9 mM NAC groups after storage for 72 h at 4 ℃. (c) JC-1 fluorescence intensity (mitochondrial membrane potential) in the control, 5, 7, and 9 mM NAC groups after storage for 72 h at 4 ℃.

The effect of NAC on mitochondrial fuction of sperm cells during chilled storage is shown in Fig 5. ROS levels in the 5 and 7 mM NAC groups were significantly lower than those in the control group (*P* < 0.001, Fig 5a). The ATP content in the 5 and 7 mM NAC groups was significantly higher than that in the control group (*P* < 0.01 and *P* < 0.001, respectively, Fig 5b), and the mitochondrial membrane potential was increased in all NAC treatment groups (5, 7, and 9 mM; *P* < 0.001, Fig 5c). These results demonstrate the protective effect of NAC during chilled storage, with 7 mM showing the greatest efficacy in preserving sperm functionality.

#### Effect of different NAC concentrations on sperm plasma membrane and acrosome integrity after chilled storage for 72 h.

**Fig 6:**
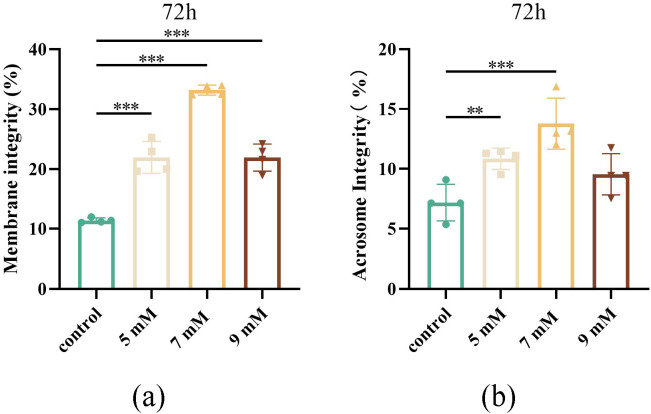
Effects of different concentrations of NAC on plasma membrane integrity and acrosome integrity of sperm cells (a) The membrane integrity of the control, 5, 7, and 9 mM NAC groups after storage for 72 h at 4 ℃. (b) The acrosome integrity of the control, 5, 7, and 9 mM NAC groups after storage for 72 h at 4 ℃.

The sperm plasma membrane is a crucial barrier maintaining the stability of the intracellular and extracellular environment, preventing leakage of cell contents and entry of toxic substances [23]. Its integrity directly reflects sperm viability. Only sperm with intact plasma membranes can maintain normal ion gradient, membrane potentials, and metabolic activity, providing energy for motility, capacitation, and fertilization. In this study, the plasma membrane integrity of the 5, 7, and 9 mM NAC groups was significantly higher than that of the control group (*P* < 0.001, Fig 6a). An intact acrosome is essential for sperm to recognize and bind to oocytes, undergo the acrosome reaction, release hydrolytic enzymes to penetrate the zona pellucida, and ultimately achieve fertilization. In this experiment, the acrosome integrity in the 5 and 7 mM NAC groups was significantly higher than that in the control group (*P* < 0.01 and *P* < 0.001, respectively, Fig 6b). Therefore, adding an appropriate concentration of NAC improved sperm acrosome integrity.

#### Effect of different NAC concentrations on sperm apoptosis after chilled storage.

**Fig 7:**
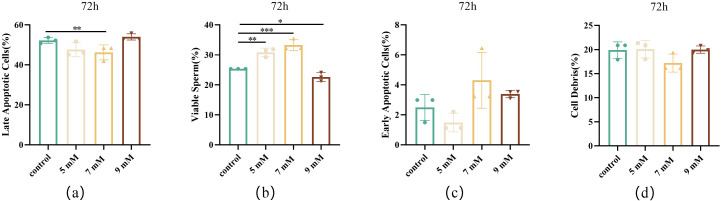
Effects of different concentrations of NAC on sperm cell apoptosis (a) The proportion of late apoptotic cells in the control, 5, 7, and 9 mM NAC groups after storage for 72 h at 4 ℃. (b) The proportion of viable cells in the control, 5, 7, and 9 mM NAC groups after storage for 72 h at 4 ℃. (c) The proportion of early apoptotic cells in the control, 5, 7, and 9 mM NAC groups after storage for 72 h at 4 ℃. (d) The proportion of cell debris in the control, 5, 7, and 9 mM NAC groups after storage for 72 h at 4 ℃.

To further investigate the protective mechanism of NAC, its effects on sperm apoptosis after 72 h of chilled storage were assessed. Fig 7 shows that there were no significant differences in early apoptosis rates or cell debris between the control and NAC-treated groups. However, the 7 mM NAC treatment demonstrated marked protective effects: viable cell counts increased substantially (*P* < 0.001, Fig 7b), while late apoptosis was significantly reduced (*P* < 0.01, Fig 7a). These results indicate that 7 mM NAC effectively inhibited sperm apoptosis induced during chilled storage.

#### Effect of different NAC concentrations on sperm antioxidant and apoptosis-related gene expression after chilled storage.

**Fig 8:**
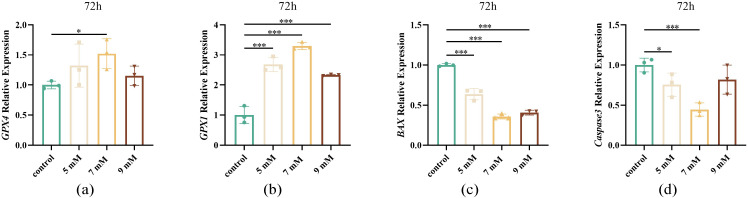
Effects of different concentrations of NAC on the expression of antioxidant and apoptosis genes in sperm (a, b) The effect of NAC on the expression levels of *GPX1* and *GPX4* in sperm after storage for 72 h at 4 ℃ (c, d) The effect of NAC on the expression levels of *BAX* and *Caspase 3* in sperm after storage for 72 h at 4 ℃.

NAC treatment significantly altered gene expression levels related to oxidative stress and apoptosis (Fig 8). The expression of antioxidant genes *GPX4* and *GPX1* was significantly upregulated (*P* < 0.05 and *P* < 0.001, respectively; Fig 8a, b), while that of pro-apoptotic markers *BAX* and *Caspase3* was downregulated in NAC- treated samples (*P* < 0.001, Fig 8c, d). These molecular changes indicate enhanced antioxidant capacity and reduced apoptotic signalling in sperm following chilled storage.

## Discussion

Semen quality, including parameters such as ejaculate volume, sperm motility, density, morphology, and acrosomal integrity, fundamentally determines fertilization capacity and pregnancy outcomes [24]. Previous research by Yang et al. has shown that adding 1 mM LC and 0.5 mM NAC significantly mitigated the detrimental effects of low-temperature storage on sperm motility, progressive motility, kinetic parameters, plasma membrane integrity, and acrosome integrity in boar spermatozoa [25]. Our results confirm these observations: compared with the untreated control group, sperm motility in NAC-treated groups was significantly improved at 24, 48, and 72 h storage. This enhancement is likely attributed to NAC#39;s ability to reduce lipid peroxidation, thereby preserving plasma and mitochondrial membrane integrity. Such structural preservation may help maintain ATP production for flagellar movement, thereby supporting progressive motility. Sperm motility classifications include progressive and non-progressive movement (only lateral head displacement), and immobility [26]. Our data show that NAC supplementation significantly increases progressive motility while decreasing the number of immotile sperm.

Reproductive tissues employ multi-layered antioxidant defense mechanisms, including enzymatic components (SOD, CAT, GPX) [27] and non-enzymatic molecules (glutathione, vitamins, polyphenols), to protect sperm from ROS-induced damage. Superoxide dismutase catalyzes the conversion of superoxide anions into hydrogen peroxide and oxygen, thereby reducing oxidative stress and exerting an anti-inflammatory effect [28]. Subsequently, catalase breaks down hydrogen peroxide into water and oxygen, eliminating its cytotoxicity. In mammalian systems, glutathione peroxidase provides an alternative H₂O₂ detoxification pathway, producing water and oxidized glutathione (GSSG). GSSG reductase then regenerates GSH from GSSG, completing a protective cycle [29]. Glutathione biosynthesis is highly dependent on cysteine availability and glutamate-cysteine ligases’ activity [30]. Total antioxidant capacity serves as a comprehensive biomarker of oxidative stress [31]; its decrease indicates increased oxidative damage and a higher risk of DNA fragmentation [32]. L-cysteine supplementation has been shown to effectively protect boar sperm stored at 4–5°C [33]. Our results indicate that 7 mM NAC optimally enhances the activity of the antioxidant enzyme activity. SOD levels were significantly increased, while CAT activity reached its maximum in this treatment, indicating a stronger ability to break down hydrogen peroxide. This powerful antioxidant response may reduce oxidative damage and help maintain sperm motility and fertilization potential [34]. GSH-Px neutralizes lipid peroxides and prevents membrane damage using glutathione [35], and both GSH-Px and total GSH levels were significantly increased in the 7 mM NAC group.

ROS, comprising superoxide anions, hydroxyl radicals, hydrogen peroxide, and singlet oxygen—arise primarily from mitochondrial respiration [36]. Oxidative stress induces sperm damage through multiple mechanisms: including lipid peroxidation, DNA strand breaks, and protein oxidation, ultimately leading to sperm dysfunction and decreased motility [37]. Our results showed that the 7 mM NAC treatment significantly reduced ROS levels compared with the other groups, which coincided with the highest semen quality parameters observed in this study. In addition, ATP levels were markedly increased in the 7 mM group, suggesting that NAC helps preserve mitochondrial activity and energy metabolism. These findings indicate that an appropriate concentration of NAC may improve sperm function by reducing oxidative stress and maintaining cellular energy supply.

Structural integrity is the fundamental material basis for sperm motility and fertilization function. When sperm cell structure is compromised, sperm may lose their motility and fertility. The integrity of the plasma membrane also plays an important role in maintaining normal physiological functions of sperm cells. In this study, the plasma membrane integrity of the sperm cells of Maguan hornless goat was the best in the 7 mM NAC group, which is consistent with previous reports [38]. Previous studies have shown that antioxidant supplementation (e.g., catalase and glutathione) in semen diluents improves plasma membrane integrity following cryopreservation, supporting the role of antioxidants in protecting sperm membranes. This result is consistent with the highest sperm motility observed in the 7 mM NAC group. The function of mitochondria directly determines the energy supply and flagella motility of sperm, and its membrane potential can be used as a direct embodiment of mitochondrial energy metabolism, which has become an extremely important index to evaluate sperm quality. In this experiment, mitochondrial membrane potential in the 7 mM NAC group was significantly higher than that in the control group. The main reason being that due to the protection provided by NAC in the 7 mM NAC group, ROS cannot attack the endometrial phospholipids of sperm cells, which leads to a decrease in permeability [39] and cell content leakage, resulting in a higher mitochondrial membrane potential in the 7 mM NAC group than that in the control group. Acrosome integrity reflects the structural and functional integrity of the sperm acrosome and is closely associated with sperm fertilization potential [40]. In the present study, acrosome integrity was significantly higher in the 7 mM NAC group compared with the other groups. This suggests that 7 mM NAC can effectively protect the acrosomal membrane. Excessive ROS can directly attack membrane lipids, leading to lipid peroxidation, abnormal membrane fluidity, and structural disruption of the acrosome. Similar findings have been reported in previous studies. Sun et al. demonstrated that supplementation of α-lipoic acid (ALA), another antioxidant, significantly improved sperm acrosome integrity in ram semen during storage by reducing oxidative stress [41]. Consistent with these findings, the present study also showed that NAC significantly reduced ROS levels and improved acrosome integrity. These results confirm the protective effect of NAC against ROS-induced lipid peroxidation and membrane damage.

Apoptosis is a programmed cell death process [42] that plays an important role in regulating sperm quality and functional integrity. The 7 mM NAC treatment exhibited the lowest level of apoptotic cells, likely due to effective ROS scavenging, consistent with previous reports on schisandrin B effects in boar semen [43]. Reduced early-stage apoptosis may be associated with anti-apoptotic proteins such as Bcl-2 and Bcl-xL, which prevent mitochondrial pore formation and inhibit the release of cytochrome c [44].In contrast, late-stage apoptosis proceeds via the mitochondrial pathway, where stress signals trigger cytochrome c release and active the caspase cascade [45], ultimately leading to DNA damage and cell death [46]. In the present study, the 7 mM NAC group also exhibited minimal late-stage apoptosis. Similar antioxidant-mediated anti-apoptotic effects have been reported for other compounds. For example, Mito-TEMPO supplementation significantly inhibited sperm apoptosis during liquid preservation [47]. During chilled storage, oxidative stress induces lipid peroxidation and apoptosis. Like melatonin [48] and curcumin [49], NAC enhances the transcription of antioxidant-related genes while suppressing apoptotic pathways, providing molecular-level evidence for its protective mechanisms [50]. This regulation is likely mediated by its ability to increase intracellular GSH levels, thereby reducing oxidative stress. Furthermore, NAC has been shown to directly activate the Nrf2 signaling pathway via thiol modification of Keap1, involving disulfide bond formation between its cysteine residues [51]. This activation leads to the nuclear translocation of Nrf2 and subsequent upregulation of phase II antioxidant enzymes such as SOD, CAT, and GPX [52], which further strengthens the cellular defense against oxidative damage. In summary, NAC supplementation provides effective protection to Maguan hornless goat sperm during chilled storage by enhancing antioxidant capacity and reducing mitochondrial-mediated apoptosis. These effects contributed to improved sperm survival and functional integrity under the experimental conditions. Therefore, appropriate NAC supplementation may serve as a useful strategy for improving semen preservation in this breed and may provide a reference for future studies on germplasm preservation of rare goat genetic resources.

## Conclusion

Collectively, supplementation with 7 mM NAC significantly enhanced antioxidant gene expression and antioxidant enzyme activity in Maguan hornless goat sperm after 72 h of chilled storage. This was associated with reduced oxidative stress, improved plasma membrane and acrosome integrity, decreased apoptosis, and increased mitochondrial membrane potential. These findings demonstrate that 7 mM NAC improves the antioxidant defense capacity and overall semen quality of Maguan hornless goat sperm during chilled storage. These results provide valuable insights for optimizing chilled semen preservation in goats and may support future applications in reproductive management and genetic resource conservation. However, further artificial insemination trials are needed to determine whether these improvements in semen quality translate into enhanced fertility and pregnancy outcomes.

## Supporting information

S1 Data S1-Data. (XLSX)

S2 File S2-Mean and standard deviation. (XLSX)
